# Optimized wavelength selection for eggplant seed vitality classification using information acquisition techniques

**DOI:** 10.3389/fpls.2025.1584269

**Published:** 2025-06-03

**Authors:** Bing Yang, Xuyang Liu, Dongfang Zhang, Xiaofei Fan, Bo Peng, Jun Zhang

**Affiliations:** ^1^ College of Mechanical and Electrical Engineering, Hebei Agricultural University, Baoding, China; ^2^ College of Economics and Management, Hebei Agricultural University, Baoding, China; ^3^ State Key Laboratory of North China Crop Improvement and Regulation, Hebei Agricultural University, Baoding, China; ^4^ College of Horticulture, Hebei Agricultural University, Baoding, China

**Keywords:** information acquisition techniques, wavelength selection, hyperspectral, eggplant seed, vitality classification

## Abstract

Eggplant seed vigor is a crucial indicator of its germination rate and seedling growth quality. In response to the need for efficient and nondestructive assessment methods, this study explores the use of hyperspectral imaging combined with advanced feature selection and classification algorithms to evaluate eggplant seed viability. Hyperspectral imaging was employed to collect spectral data from eggplant seeds, covering 360 bands within a wavelength range of 395.24–1008.20 nm. The seeds underwent microwave heating and constant-temperature water bath aging treatments. Data preprocessing involved three techniques: Multiplicative Scatter Correction (MSC), Savitzky–Golay (SG) smoothing, and Standard Normal Variate (SNV) transformation. An Enhanced Information Acquisition Optimization (EIAO) algorithm was proposed for feature selection, which successfully identified a minimal set of 23 key wavelengths. Seed vigor classification models were developed using Extreme Learning Machine (ELM), Random Forest (RF), and Support Vector Machine (SVM).The optimal classification accuracies achieved were 90.0% for ELM, 91.45% for RF, and 90.5% for SVM. The MSC-EIAO-RF model demonstrated the best performance, achieving an accuracy of 91.45%, which is 9.04% higher than the MSC-IAO model (82.41%).Validation on four UCI datasets further confirmed the EIAO algorithm's superiority over conventional feature selection methods. These results verify the robustness and generalizability of hyperspectral imaging combined with EIAO for nondestructive seed viability detection, offering an intelligent and efficient solution for seed quality assessment.

## 1 Introduction

Eggplant is a perennial herb widely cultivated in warm tropical and subtropical regions worldwide. Due to its high mineral content and low caloric value, it is considered one of the healthiest fruits and vegetables (Abubakar et al., 2023; Asafew and Chandravanshi, 2021; Yamaguchi et al., 2019). Eggplant seeds are flat, shiny, and either reddish-black or yellow, with fine lines on the seed coat but no hairs, exhibiting strong vitality. Seed quality is commonly assessed based on germination potential and vigor (Xing et al., 2023). Among these, seed vigor is the most crucial indicator of seed quality, as it directly influences seedling germination rates and the overall health of the plant (Ventura et al., 2012). For seeds stored over extended periods, especially those used for breeding or conserving genetic diversity, seed vigor remains a prominent research focus (De Vitis et al., 2020). Even under optimal storage conditions, seed viability inevitably declines over time, a phenomenon referred to as seed aging (Ebone et al., 2019). Traditional methods for assessing seed vigor, such as conductivity and red ink tests, are often complex, inefficient, and somewhat destructive. These methods play a critical role in determining the quality grade and shelf life of seeds, making them essential for evaluating seed vitality and longevity (Wang et al., 2021a). Therefore, developing more advanced and innovative testing methods to enhance the efficiency of existing seed testing technologies has become a critical area of research.

In recent years, Hyperspectral imaging (HSI) technology has garnered widespread attention in seed quality assessment due to its non-destructive and rapid characteristics. By providing spatial and spectral information related to plant and biochemistry, the technology has excellent capabilities in seed variety classification and grading, seed viability and damage detection, and seed composition determination (Feng et al., 2019). In seed classification, researchers have combined hyperspectral imaging technology in combination with the firefly optimization algorithm to optimize deep learning parameters, successfully achieving vitality detection of sweet corn seeds (Wang and Song, 2024). Huang et al. employed HSI to classify corn seeds from four different years and developed a classification model based on the average spectral features of the seeds, utilizing the least squares support vector machine (LSSVM) (Huang et al., 2016). Wang et al. employed near-infrared hyperspectral imaging (NIR-HSI) technology to study the maturity classification of corn seeds. They extracted the average spectra from the embryo side (T1) and the endosperm side (T2) of the seeds, and calculated the average spectrum of both sides (T3). Principal component analysis (PCA) was applied to select the characteristic wavelengths. When T1 and T2 were used as inputs for the optimal model, the classification accuracy reached 98.7% and 100%, respectively (Wang et al., 2021b). In classifying camellia oil grades, researchers collected hyperspectral images of camellia oil samples from three different grades. The successive projections algorithm (SPA) and competitive adaptive reweighted sampling (CARS) were employed to extract spectral and texture features. Subsequently, the genetic algorithm (GA) was used to optimize the kernel function of the support vector machine (SVM), along with its corresponding kernel function parameters and penalty factors. The results demonstrated that the model classification performance was best when GA was used to optimize the SVM (Gu et al., 2024). These studies highlight the significance and potential of hyperspectral imaging (HSI) in seed vigor detection. By providing spatial and spectral information related to plants and their biochemical characteristics, HSI holds great promise for the non-destructive assessment of seed vigor and other key quality parameters.

However, wavelength selection remains a critical challenge in applying hyperspectral imaging technology to seed identification (Huang et al., 2024; Huang and Xia, 2023). Selecting characteristic wavelengths can enhance data processing efficiency, highlight important features, and improve model robustness (Huang et al., 2022; Ong et al., 2023). In the classification of rapeseed maturity, the continuous projection algorithm (SPA), competitive adaptive reweighted sampling (CARS), and interval variable iterative spatial shrinkage (IVISSA) are combined to select spectral wavelengths. The results indicate that the algorithm combining SPA and IVISSA achieves an accuracy of 97.86% (Feng et al., 2024). In addition to conventional characteristic wavelength selection methods, biologically inspired meta-heuristic algorithms have emerged as effective tools for hyperspectral band selection. A representative application can be found in the research on chlorophyll content prediction in Chinese cabbage using hyperspectral technology. In this study, the reflectance data underwent comprehensive preprocessing through standard normal variate (SNV) transformation, Savitzky-Golay (SG) smoothing, and second derivative (2D) analysis. Subsequently, a genetic algorithm (GA) was employed to identify optimal spectral characteristic bands, which were then utilized to construct a sophisticated one-dimensional convolutional neural network (1D-CNN) prediction model (Zhang et al., 2023). For the identification of wheat grain varieties, the interval random frog (iRF) algorithm, an advanced wavelength selection method, was implemented to optimize the spectral wavelength intervals through an iterative jumping mechanism (Que et al., 2023). Although existing studies have proposed several effective wavelength selection methods, such as the integration of convolutional neural network architectures with traditional feature selection algorithms (e.g., SPA, CARS, and IVISSA), these methods have demonstrated significant improvements in classification accuracy. However, they still exhibit certain limitations.

Traditional methods may fail to fully capture key features in spectral data and tend to be slower when handling large datasets with many redundant features. To address these challenges, this study presents a novel wavelength selection algorithm (EIAO) based on information acquisition optimization, designed to enhance the efficiency of spectral data processing and improve classification accuracy. By processing and optimizing the hyperspectral data of eggplant seeds, the EIAO algorithm effectively selects the most informative spectral bands, offering an innovative and efficient solution for the non-destructive detection of seed vigor.

## 2 Materials and methods

### 2.1 Data collection

**Figure 1:**
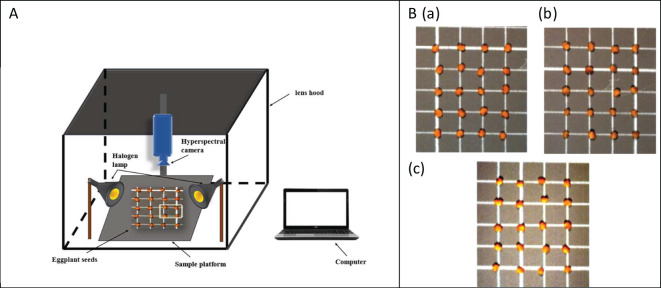
**(A)** Hyperspectral data acquisition system. **(B)** Artificially aged eggplant seed samples (a) control group without any treatment (b) water bath group (c) microwave group.

In the experiment, four varieties of eggplant with complete shape and uniform size were selected for artificial aging treatment, with three experimental groups set up (**Figure 1B**), namely the microwave group, the water bath group, and the control group with no treatment. Each aging treatment was repeated three times to eliminate the influence of seed variability and treatment-induced effects, ensuring that no visible changes appeared on the seed surface. After treatment, the seeds were air-dried at room temperature to restore their original weight. The experiment utilized an HG101 portable hyperspectral imaging system (Zhongchuan Optoelectronics Precision Machinery Co., Ltd., Beijing), as shown in **Figure 1A**. Hyperspectral data were collected from these eggplant seed samples using a hyperspectral camera, which covers a spectral range of 395–1008 nm with 360 bands, and includes components such as a halogen lamp, a sample board, and a light shield. To ensure clear and accurate imaging, the camera lens-to-sample distance was fixed at 30 cm, and the exposure time was set to 15 milliseconds. Prior to capturing hyperspectral images, a whiteboard calibration was performed to correct for any potential lighting inconsistencies. After imaging, each seed, categorized by its aging level, was sealed in a bag and numbered for subsequent identification and analysis of seed vigor. Following these procedures, the spectral images of the samples were successfully captured and stored.

### 2.2 Software and performance evaluation

This study used ENVI (version 5.6, Harris Corporation, USA) to extract the reflectance of eggplant seeds and MATLAB (version 2023a) for characteristic wavelength selection and classification modeling tasks. The dataset was divided into an 8:2 ratio, resulting in 800 training samples and 200 test samples. When analyzing the results of data dimensionality reduction, the fitness score defined in Eq. (5) was used to evaluate the quality of potential optimal feature subsets. Accuracy represents the classification performance of the feature subset on the classifier, while the number of dimensions of the feature subset indicates how many important features contribute to the classification.

In the EIAO framework, the iteration count *K* was set to 50. The parameter settings for other comparison algorithms are provided in **Table 1**. These parameters are essential for guiding the algorithm’s search process, balancing exploration and exploitation, and ensuring efficient convergence toward the optimal solution.

**Table 1:** Parameter setting.

Algorithms	Parameter values
EIAO	v, β, γ, and δ random numbers over [0,1] are set as the literature [15]
IAO	v, β, γ, and δ random numbers over [0,1] are set as the literature [15]
PSO	c1=c2 = 2, ω=1
CSA	α=δ=0.1 , r1 = 10, U=0.00565, ω=0.005
SO	Q=0.25, T=0.6, c1 = 0.5, c2 = 0.05, c3 = 2
SCA	a=2

### 2.3 Data analysis methods

#### 2.3.1 Data preprocessing

Spectral data often contains noise from the environment and instruments. Preprocessing the spectral data can enhance the accuracy of the model (Chen et al., 2024). In this study, we applied three preprocessing methods: MSC, SG, and SNV.

#### 2.3.2 Feature extraction

Hyperspectral data often contains redundant information due to the large number of spectral bands. Data dimensionality reduction can help select effective characteristic wavelengths, thereby reducing model computation and improving operational efficiency. In this study, the Enhanced Information Acquisition Optimization (EIAO) algorithm is used to extract characteristic wavelengths, with improvements made to the original Information Acquisition Optimization algorithm. A detailed explanation of the algorithm is provided below.

**Figure 2:**
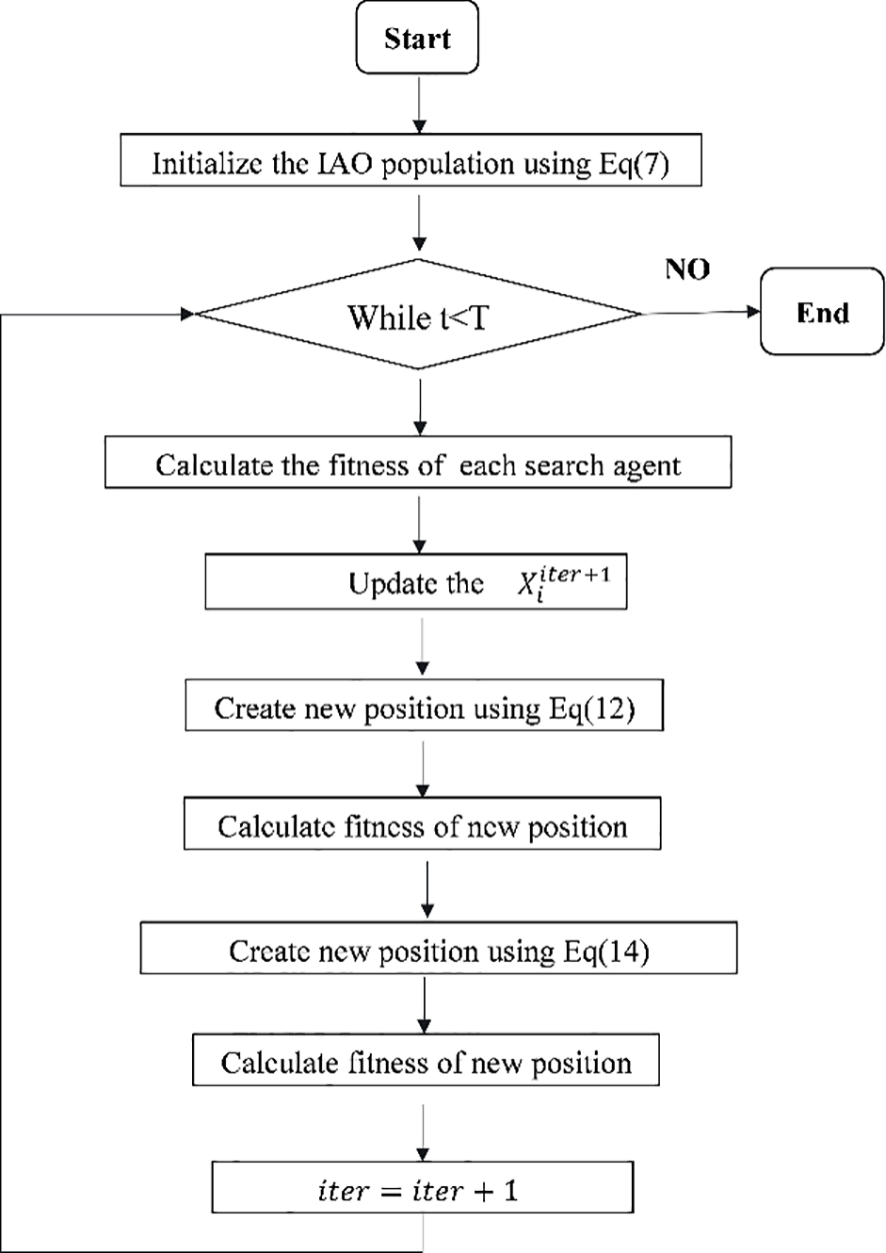
The flowchart of the proposed algorithm EIAO.

The original Information Acquisition Optimization Algorithm (IAO) algorithm was recently proposed (Wu et al., 2024). The basic idea is to simulate the ability of humans to process massive amounts of information. It includes three steps: collecting information, filtering and evaluating information, and analyzing and organizing information. When EIAO is applied to the feature selection problem, We define a population of N human individuals as a two-dimensional matrix X = { $x_{1}\;,\ldots \;,x_{i}\;,\;\ldots \;,x_{N}$ }={ $x_{j}^{i}|\;1\leq i\;\leq N,\;1\leq j\;\leq D$ . $x_{j}^{1}$ represents the individual at the first position in the jth dimension. $x_{j}^{i}$ represents the i-th individual in the j dimension. When IAO is used for feature selection problems, all solutions are restricted to binary values. That is, $x_{j}^{i}$ ∈[0,1]. At this time, $x_{j}^{i}$ represents the j-th feature in the i-th seed sample. If $x_{j}$ =1, it means that the feature is selected. The feature selection framework based on the EIAO algorithm is shown in **Figure 2**.

**Figure 3:**
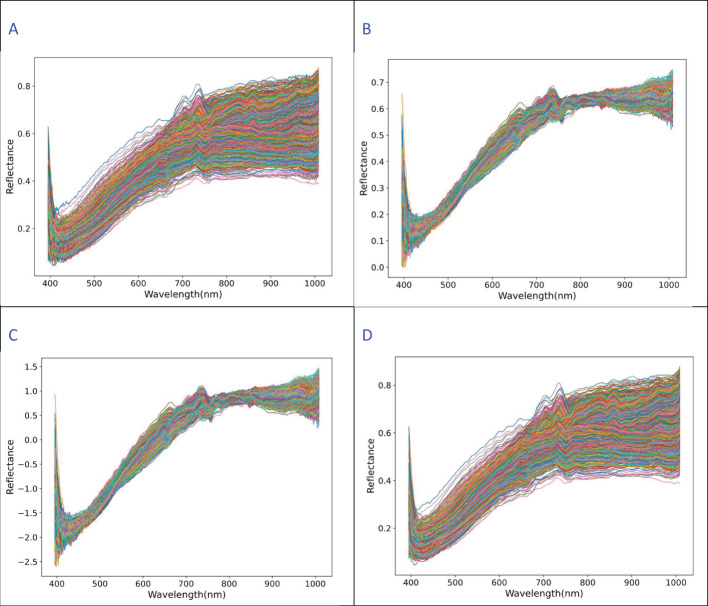
Hyperspectral reflectance and preprocessing. **(A)** The raw average reflectance **(B)** MSC preprocessing **(C)** SNV preprocessing **(D)** SG smoothing Preprocessing image.

As shown in **Figure 3**, the first step of EIAO is population initialization. Building on the initialization method of the original algorithm, this study selects 10% of the population size as the number of elite individuals and applies chaotic reverse learning to the selected elites. Elite chaotic reverse learning is an initialization technique that combines elite strategies, chaotic disturbances, and reverse learning. It is primarily used to enhance the diversity and exploration ability of the population.

The expression for the initial state is shown in Equation 1.


(1) $$x_{j}^{i}\left(k\right)=\left(ub_{j}\left(k\right)-lb_{j}\left(k\right)\right)*\theta^{k}+lb_{j}\left(k\right)$$

The expression of logistic mapping is


(2) $$\theta^{k+1}=\;\mu \theta^{k}\left(1-\theta^{k}\right)$$

According to Equation 2, chaos mapping is a nonlinear dynamic system that generates random numbers with unique dynamic characteristics. These random numbers are distinguished by their non-repeatability, accessibility, regularity, and unpredictability (Zhang and Feng, 2018). The reverse learning strategy is an intelligent calculation method introduced (Tizhoosh, 2005) in 2005. It has been widely applied in other group-based intelligent optimization algorithms to enhance their search performance. Building on the effectiveness of reverse learning strategies, Wang et al. introduced general reverse factors and proposed the concept of general reverse learning strategies (Wang et al., 2011). Li et al. introduced the concept of elite learning and proposed an elite reverse learning strategy based on general reverse learning strategies. Experimental results demonstrated that the elite reverse learning strategy outperforms the general reverse learning strategies (Li, 2012).

Since the band selection of the spectrum belongs to the feature selection of binary problems, it is necessary to convert the EIAO continuous form into the EIAO-FS binary discrete form. Thus converted from Equation 1 to Equation 3. The expression of T in Equation 3 is as shown in Equation 4.


(3) $$x_{j}^{i}\left(k\right)=\{\begin{array}{c} 1,\;T\left(x_{j}^{i}\left(k\right)\right)\geq C\\ 0,T\left(x_{j}^{i}\left(k\right)\right)<C \end{array}$$

(4) $$T\left(x_{j}^{i}\left(k\right)\right)=\frac{1}{1+exp^{-x_{j}^{i}\left(k\right)}}$$

Fitness calculation


(5) $$fitness=\alpha \rho_{R}\left(Y\right)+\beta \frac{\left|K\right|}{\left|N\right|}$$

As shown in Equation 5, among them, $\rho_{R}\left(Y\right)$ represents the error rate of the KNN classifier, and α and β meet the α∈ [0, 1] and α+ β = 1, respectively. |K| is the number of selected features, |N| is the number of data concentration original features. Under normal circumstances, the smaller the adaptation value, the better.

K-NN is a non-parametric supervised classification learning algorithm. The category of a new sample is determined by the K nearest training samples to the new sample (Pernkopf, 2005). Here, the K−NN model is used as a classification method to evaluate the features generated by EIAO, where K = 3.

The first stage involves collecting information and establishing an initial information system. The mathematical model is expressed as Equation 6.


(6) $$x_{i}^{iter+1}=x_{i}^{iter}+\vartheta \times \left(x_{i}^{r1}-x_{i}^{r2}\right)$$

Where iter represents the current iteration number, ϑ is a random number between [0,1], and $x_{i}^{r1}$ and $x_{i}^{r2}$ are two information bodies randomly generated during iteration. Consider $(x_{i}^{r1}-x_{i}^{r2}$ ) as a differential variable, and refer to DE/rand/2/bin to add another differential variable $\left(x_{i}^{r3}-x_{i}^{r4}\right)$ . At this time, the information collection phase is updated through Eq (7). By adding more information bodies, more randomness and diversity are introduced during the update, thereby improving the breadth of the search. It helps to avoid premature convergence to the local optimal solution. Multiple information bodies can make the exploration of the search space more comprehensive, thereby increasing the diversity of the population. The updated first-stage mathematical model is as shown in Equation 7.


(7) $$x_{i}^{iter+1}=x_{i}^{iter}+\vartheta \times \left(x_{i}^{r1}-x_{i}^{r2}\right)+\vartheta \times \left(x_{i}^{r3}-x_{i}^{r4}\right)$$

The second stage is information filtering evaluation, the expression is as in Equation 8:


(8) $$\{\begin{array}{c} x_{i}^{iter+1}=x_{i}^{iter}-\Delta \times rand\times \left(x_{i}^{rand}-x_{i}^{iter}\right),if\;rand<0.5\\ x_{i}^{iter+1}=x_{i}^{iter}+\Delta \times rand\times \left(x_{i}^{rand}-x_{i}^{iter}\right),otherwise \end{array}$$

Among them, $\Delta =\frac{\cos\left(\frac{\pi }{2}\times \sqrt{\left|\Gamma \right|}\right)}{\Xi }$ represents the error caused by subjective factors in filtering and evaluating information, and Ξ is the subjective influencing factor, which reflects the individual’s less correct judgment on information processing due to preferences, experience, emotions, etc. Its value is calculated by the following Equation 9:


(9) $$\Xi =2\times \mathrm{mod}\left(3.648\times \nu \times \left(1-\beta \right)\times \left(a\cos\left(\gamma \times 10^{4}\right)\right)\right),1)$$

ν , β , and γ are random numbers generated between [0, 1]. Γ is defined as a reliability factor, which characterizes the ability of the algorithm to optimize its behavior by self-adjusting the quality of information at different stages. This design enhances the adaptability and flexibility of the algorithm. Γ is calculated by the following Equation 10.


(10) $$\Gamma =\sin\left({\left(\frac{\pi }{4}\right)}^{\frac{iter}{Max_{iter}}}\right)+\emptyset +\frac{\log_{10}\frac{iter}{Max_{iter}}}{8}$$

where Φ is the information quality factor, the expression is as in Equation 11.


(11) $$\emptyset =\cos\left(2\times \delta +1\right)\times \left(1-\frac{iter}{Max_{iter}}\right)$$

From Eq (8)-Eq (11), it can be seen that the iterative update is mainly determined by the difference between the current individual state $x_{i}^{iter}$ and the randomly selected reference point $x_{i}^{rand}$ . Although this update method introduces randomness, its search range and direction are limited and lack other dynamic adjustment mechanisms. Therefore, in complex multi-peak functions, it may not be able to effectively jump out of the local optimum. To avoid the above problems, we introduce the sine-cosine optimization algorithm (SCA) and update by the following formula (Equation 12). r5 is a random number between [0,2Π].


(12) $$\{\begin{array}{c} x_{i}^{iter+1}=x_{i}^{iter}-\Delta \times \sin\left(r5\right)\times rand\times \left(x_{i}^{rand}-x_{i}^{iter}\right),if\;rand<0.5\\ x_{i}^{iter+1}=x_{i}^{iter}+\Delta \times \cos\left(r5\right)\times rand\times \left(x_{i}^{rand}-x_{i}^{iter}\right),otherwise \end{array}$$

The final stage is to identify valuable information from the filtered data and increase the likelihood of obtaining the optimal information set. The mathematical model is expressed as Equation 13.


(13) $$\left\{ \begin{array}{l} x_{i}^{iter+1}=x_{i}^{best}\times \cos\left(\frac{\pi }{2}\times \sqrt{{\text{Λ}}^{\frac{1}{3}}}\right)-\epsilon \times \left(\frac{1}{D}\sum_{i=1}^{d}x_{i}^{best}-x_{i}^{best}\right)\;\;\;\;\;if\;\emptyset \geq 0.5\\ x_{i}^{iter+1}=x_{i}^{best}\times \cos\left(\frac{\pi }{2}\times \sqrt{{\text{Λ}}^{\frac{1}{3}}}\right)-\;0.8\times \left(\begin{array}{c} \zeta \times \kappa \times \frac{1}{D}\sum_{i=1}^{d}x_{i}^{best}-\\ \left(2\times \omega -1\right)\times x_{i}^{best} \end{array}\right)\;,otherwise \end{array}\right.$$

Where $\Lambda =2^{\left(\sqrt{\left|\Gamma \right|}-2\right)}$ , represents the control factor for analyzing and organizing information. The update formula of Eq (13) mainly relies on the local neighborhood search of the current $x_{i}^{best}$ optimal solution, combined with some dynamic adjustment factors. Although it can speed up the convergence, it may easily fall into the local optimum due to the lack of long-distance jumps. The addition of Levy flight allows individuals to make long-distance jumps with a certain probability. This jump helps to discover new potential optimal solutions, thereby balancing the ability of global exploration and local development. Updated by Eq (14).


(14) $$\left\{ \begin{array}{l} x_{i}^{iter+1}=x_{i}^{best}\times \cos\left(\frac{\pi }{2}\times \sqrt{{\text{Λ}}^{\frac{1}{3}}}\right)-\epsilon \times \left(\frac{1}{D}\sum_{i=1}^{d}x_{i}^{best}-x_{i}^{best}\right)+Levy,\;\;if\;\emptyset \geq 0.5\\ x_{i}^{iter+1}=x_{i}^{best}\times \cos\left(\frac{\pi }{2}\times \sqrt{{\text{Λ}}^{\frac{1}{3}}}\right)-\;0.8\times \left(\begin{array}{c} \zeta \times \kappa \times \frac{1}{D}\sum_{i=1}^{d}x_{i}^{best}-\\ \left(2\times \omega -1\right)\times x_{i}^{best} \end{array}\right)+Levy,otherwise \end{array}\right.$$

Levy flight is a random walk mechanism (Li et al., 2022) that enables large jumps from a local position with a high probability. The probability density distribution of Levy flight is characterized by sharp peaks, asymmetry, and heavy tails. Its movement pattern alternates between frequent short-distance jumps and occasional long-distance leaps, allowing it to escape local optima and expand the search area. Metaheuristic algorithms, inspired by natural processes, are used to solve NP-hard problems. Levy flight can serve as an operator in these metaheuristic search algorithms.

At the end of the iteration, we try to add the Laplace operator. Deep and Thakur proposed the original Laplace operator idea in 2007 (Deep and Thakur, 2007), the idea is to generate children $y_{1}=\left(y_{1}^{1},y_{1}^{2},\cdots ,y_{1}^{m}\right)$ and $y_{2}=\left(y_{2}^{1},y_{2}^{2},\cdots ,y_{2}^{m}\right)$ from the parent $x_{1}=\left(x_{1}^{1},x_{1}^{2},\cdots ,x_{1}^{m}\right)$ , and the two pairs of children are generated in a symmetric way around the position of their parents.

The random distribution of Laplace $l_{i}X$ is as Equation 15:


(15) $$l_{i}=\{\begin{array}{c} p-q\log_{e}\left(u_{i}\right),u_{i}\leq \frac{1}{2}\\ p+q\log_{e}\left(u_{i}\right),u_{i}>\frac{1}{2} \end{array}$$

Among them, $u_{i}$ is randomly and uniformly distributed on [0, 1]; p , q>0 represent the position and measurement parameters respectively. The resulting descendant relationship expression is as shown in Equation 16:


(16) $$y_{1}^{i}=x_{1}^{i}+l_{i}\left|x_{1}^{i}-x_{2}^{i}\right|,y_{2}^{i}=x_{2}^{i}+l_{i}\left|x_{1}^{i}-x_{2}^{i}\right|$$

As an effective local search technique, some researchers have combined Levy flight (LX) with the Harris Hawk Optimization (HHO) algorithm, demonstrating its unique and superior performance on certain complex optimization problems (Nasab and Abualigah, 2024). This shows us the potential of this local search.

#### 2.3.3 Performance evaluation

After obtaining the optimal feature subset using the EIAO algorithm, we constructed three classification models to evaluate their classification performance.

The extreme learning machine (ELM) is an advanced one-way feedback neural network algorithm based on a feedforward neural network (Huang et al., 2006). When using the ELM to build the seed activity discrimination model, the activation function employed is the S-shaped function. The number of neurons in the extreme learning machine is set within the range of 30 to 100, with a step size of 10. Simultaneously, the number of hidden layer neurons is adjusted to determine the optimal configuration for different spectral data. ELM exhibits strong generalization ability when handling high-dimensional data and is particularly well-suited for large-scale classification tasks. Therefore, in this study, ELM was chosen to develop the seed vigor classification model, aiming to enhance both classification accuracy and processing efficiency.

RF is a decision tree ensemble model based on the bagging strategy (Breiman, 2001). RF integrates multiple decision trees, each of which is trained on the original training set. The final classification result is obtained by voting among all decision trees (Si et al., 2023). In this study, the parameter “N” of the bagging framework is set to 300, the maximum depth “M” of the decision tree ranges from 1 to 20, and the step size of the grid search is 1. RF is effective in handling datasets with high noise and complex patterns, and it can automatically assess feature importance, offering enhanced interpretability. In this study, RF was employed to evaluate the stability of seed vigor classification, particularly in addressing noise and variations in spectral data.

SVM is a classic supervised machine learning model capable of classifying both linear and nonlinear data. It is a well-established binary classification model in supervised learning (Piccialli et al., 2024). It is widely used across various fields. In this study, the SVM algorithm utilizes the radial basis function (RBF) as the kernel function, with the penalty factor ‘C’ and kernel parameter ‘G’ optimized through 5-fold cross-validation.

#### 2.3.4 Verification of the generalization capability

This section aims to evaluate the generalization capability of the EIAO algorithm. To this end, EIAO, along with five other optimization algorithms, is tested on four datasets from the UCI Machine Learning Repository. The UCI Repository, created by the University of California, Irvine, is a widely recognized collection of machine learning datasets. It includes a diverse range of datasets designed for various machine learning tasks such as classification, regression, and clustering. Each dataset typically provides details such as the dataset name, features, labels, descriptive documentation, and data formats. The Ionosphere dataset was collected using a ground-based radar system; The Arrhythmia dataset was obtained using electrocardiogram sensors that recorded cardiac signals; The Vehicle dataset contains features extracted from vehicle images using image processing techniques; The Vote dataset is based on survey records of U.S. Congressional voting behavior. These datasets are all commonly used for classification tasks. For this study, four classification datasets are selected, as summarized in **Table 2**.

**Table 2:** UCI datasets used in this study.

Dataset	Number of Samples	Number of Features	Class
Ionosphere	351	34	2
Arrhythmia	452	279	16
vehicle	846	18	4
vote	435	16	2

## 3 Results

### 3.1 Hyperspectral data preprocessing analysis

The resulting processed spectral curve is shown in **Figure 3**. The hyperspectral reflectance curve of eggplant seeds shows a clear upward trend in the 500–700 nm range, while the reflectance in the near-infrared band remains relatively flat, with high reflectance values. This is attributed to the presence of water and other tissue components in the seeds, which strongly reflect near-infrared light. The reflectance characteristics of eggplant seeds vary in this range depending on the treatment. Around 750 nm, the curve fluctuates, initially rising and then falling, due to differences in seed water content (**Figure 3A**). The changes in light source from the two halogen lamps in the hyperspectral equipment and variations in instrument response cause surface scattering of the eggplant seed samples. To correct for these effects, the multivariate scatter correction (MSC) method is applied (**Figure 3B**). Since the hyperspectral data spans a large range, the standard normal variate (SNV) method is used to eliminate large gaps in the data (**Figure 3C**). The influence of the external environment introduces errors in the original hyperspectral reflectance, which are corrected by the Savitzky-Golay (SG) smoothing filter to remove irrelevant signals and smooth the data (**Figure 3D**).

### 3.2 Feature extraction result analysis

#### 3.2.1 Comparative performance of EIAO and baseline algorithms

In this study, the Enhanced Information Acquisition Optimization (EIAO) algorithm was used to extract feature bands from the collected seed hyperspectral data. PSO (Kocak and Orkcu, 2024), Chameleon Optimization Algorithm (CSA) (Braik et al., 2023a), Snake Optimization Algorithm (SO) (Braik et al., 2023b), Sin-Cosine Optimization Algorithm (SCA) (Sun et al., 2022) and the standard IAO, as outlined in **Table 1**, are employed as baseline algorithms for comparative analysis to effectively demonstrate the overall advantages of the proposed method. Based on the fitness function in Eq. (5) from section 2.3.2, we applied the KNN model as a classification method to evaluate the quality of the feature subsets generated by each algorithm. The evaluation was carried out using the classification accuracy of the KNN model and the number of selected features.

To validate the feature selection capability of the EIAO algorithm, it was applied to four different eggplant varieties (DS_1 to DS_4) for comparison. As shown in **Table 3**, EIAO achieves the highest classification accuracy on the DS_4 dataset and selects the fewest features on DS_3. For example, in DS_4, EIAO only needs 70 features to achieve an accuracy of 97.56%, while IAO requires 90 features. In addition, the fitness function value of EIAO is significantly lower than that of other algorithms, indicating its excellent ability in balancing classification accuracy and feature redundancy. This result confirms that EIAO is highly efficient in feature selection, effectively eliminating redundant bands while maintaining excellent classification performance.

**Table 3:** Performance comparisons between EIAO and 5 baseline algorithms on Seed Spectrum datasets.

Metric	Algorithm	DS_1	DS_2	DS_3	DS_4
Accuracy (%)	IAO	90.50	85.71	72.22	95.12
	EIAO	**92.00**	**85.71**	**73.33**	**97.56**
	CSA	90.50	85.00	71.11	92.68
	SCA	90.50	85.71	71.11	95.12
	PSO	92.00	85.00	70.00	92.68
	SO	91.50	85.00	71.11	95.12
Selected features	IAO	85	50	71	90
	EIAO	**33**	**40**	**23**	**70**
	CSA	186	40	162	230
	SCA	51	60	95	150
	PSO	137	100	107	220
	SO	98	70	95	260
Best fitness function	IAO	0.0950	0.1441	0.2776	0.0498
	EIAO	**0.0816**	**0.1435**	**0.2674**	**0.0266**
	CSA	0.0992	0.1506	0.2918	0.0763
	SCA	0.0955	0.1446	0.2868	0.0495
	PSO	0.0830	0.1538	0.3008	0.0761
	SO	0.0869	0.1522	0.2894	0.0526

Bold numbers indicate the best performance among all compared methods.

At the same time, from **Table 4**, we can analyze which spectral regions of the full wavelength the selected features are mainly concentrated in. Among the selected wavelength features, the 905.70–1008.20 nm range accounted for the highest proportion (23.17%), followed by 694.92–798.40 nm (16.52%) and 490.95–589.60 nm (15.94%). These frequently selected regions were mainly located in the near-infrared range, particularly in the short-wave near-infrared region above 900 nm. This spectral region is typically associated with absorption peaks of functional groups such as O–H, N–H, and C–H, which correspond to key seed components like moisture, proteins, and starch. These components are known to change during seed aging treatments, providing a clear biochemical basis for the frequent selection of this range as effective feature wavelengths. Overall, the selected wavelengths were primarily concentrated in the information-rich near-infrared region, highlighting both the effectiveness of the feature extraction algorithm and the physical relevance of the selected features.

**Table 4:** The proportion of the feature wavelength to the full-band.

Band range (nm)	Proportion (%)
395.25–489.30	13.83%
490.95–589.60	15.94%
591.34–693.20	15.58%
694.92–798.40	16.52%
800.20–903.90	14.96%
905.70–1008.20	23.17%

#### 3.2.2 Robustness analysis of EIAO algorithm

**Figure 4:**
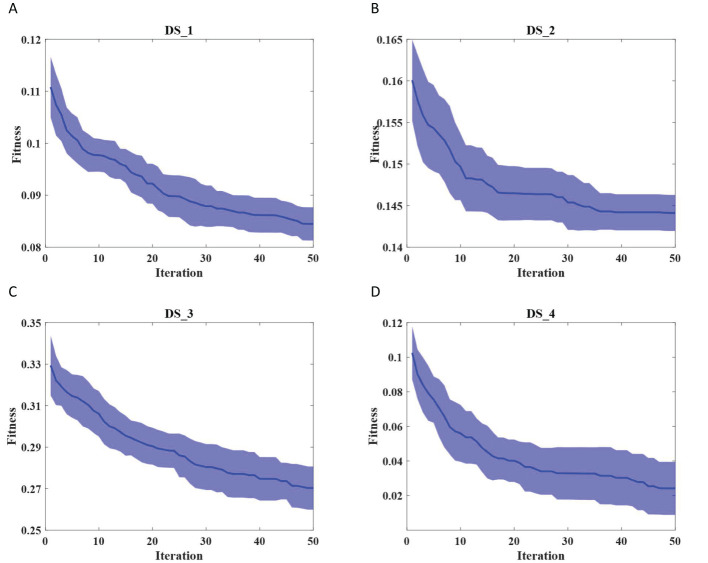
Comparison of the convergence performance of EIAO on four datasets. **(A)** DS_1, **(B)** DS_2, **(C)** DS_3, **(D)** DS_4.

**Figure 5:**
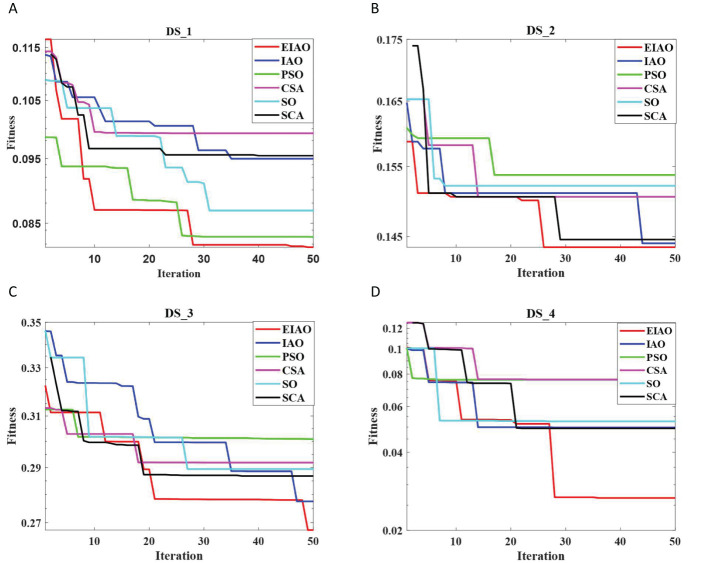
Stability analysis of EIAO and five baseline methods on four datasets. **(A)** DS_1, **(B)** DS_2, **(C)** DS_3, **(D)** DS_4.

To assess the robustness of the proposed EIAO method, we further evaluated it from two aspects. On one hand, we examined whether the convergence curve of EIAO remains stable across multiple repetitions. From **Figure 4**, we observe that the shaded region for DS_1 and DS_3 shows minimal fluctuations. The shaded area represents the standard deviation, which indicates the range of variation in the fitness values at different iterations. Standard deviation is a measure of the dispersion of data, with a larger value indicating greater differences between data points, and a smaller value indicating more concentrated data with less variation. On the other hand, **Figure 5** examines whether the EIAO algorithm quickly converges to the optimal solution during iterations. By comparing with other algorithms, we can see that EIAO not only provides better solutions than the other five algorithms, but also converges significantly faster.

#### 3.2.3 Modeling and analysis based on feature extraction

Although the analysis of four independent eggplant varieties confirmed the feature selection capability of EIAO, in practical applications, seeds are often stored in a mixed form consisting of multiple varieties. Therefore, this section combines the data from the four varieties to assess the practicality of EIAO in more complex scenarios. To evaluate the effect of feature selection on the classification model, the wavelength subsets selected by EIAO were input into the ELM, RF, and SVM models, and the results were compared with those obtained using the full wavelength data. Accuracy was used as the evaluation metric for the models. In this section, we compare the classification accuracy of three classifiers (ELM, RF, and SVM) using three sets of spectral data: raw data, preprocessed data, and data obtained after wavelength selection. This comparison aims to evaluate the effects of data preprocessing and feature selection on classification performance, providing insights into how these factors influence the overall model efficacy.

Based on the accuracy evaluation coefficient presented in **Table 5**, the following analysis can be made: First, when comparing different preprocessing methods, SG-EIAO and SNV-EIAO demonstrate the most significant optimization effects. These methods have achieved consistently favorable results across all classifiers, particularly on the test set, where the accuracy has reached 90% and 90.5%, respectively. This suggests that combining EIAO with spectral preprocessing methods can further enhance classifier performance and improve model classification accuracy across different datasets. Second, the improvement effects of EIAO optimization vary across different classifiers. In the ELM and RF models, accuracy significantly improved after optimization, especially on the test set. In the SVM model, although the improvement in test accuracy is relatively stable, its overall performance is not as high as that of the RF and ELM models. Furthermore, it was observed that, in the SVM model, the accuracy after MSC preprocessing with IAO and EIAO wavelength selection decreased notably, particularly for MSC-EIAO, where the test accuracy dropped to only 73%, significantly lower than other models. This may be attributed to the possibility that certain features, valuable to the SVM model, were discarded during feature extraction, leading to the loss of key information and a subsequent reduction in test accuracy.

**Table 5:** Accuracy of full and feature wavelength classification model.

Methods	Training accuracy (%)			Test accuracy (%)		
	ELM	RF	SVM	ELM	RF	SVM
RAW	91.87	94.87	87.22	89	83.50	87.50
MSC	92.87	96.12	86.50	85.50	87.50	85.50
SG	92	94.25	87.37	87.50	84	85
SNV	91	96.12	86.62	89.50	87	85.50
MSC-IAO	91.36	96.25	76.09	87	90.45	73.50
SG-IAO	92.49	93.87	86.50	88	82.41	84
SNV-IAO	92.61	95.87	90.86	87.50	86.93	88
MSC-EIAO	92.74	95.87	74.50	89	**91.45**	73
SG-EIAO	92.99	93.37	83.37	**90**	87.43	80.50
SNV- EIAO	92.24	95.37	90.25	**90**	89.44	**90.50**

Bold numbers indicate the best performance among all compared methods.

To further evaluate the performance of the classification models, additional metrics including precision, recall, and F1-score were introduced alongside overall accuracy. As a key indicator of a model, the F1-score effectively reflects the balance between precision and recall across different classes. As shown in **Table 6**, among the three classifiers, the Random Forest (RF) model achieved the best overall performance, with the highest F1-score of 91.45% under the MSC-EIAO method. The Support Vector Machine (SVM) model also performed well under the SNV-EIAO method, reaching a test accuracy of 90.50% and an F1-score of 90.49%. However, SVM was found to be more sensitive to feature selection strategies, with its performance significantly declining under methods such as MSC-EIAO. Overall, the MSC-EIAO method demonstrated the greatest potential among all preprocessing and feature selection strategies. It significantly improved the performance of the RF model, showed stable effectiveness in the ELM model, but may suppress performance when applied to SVM.

**Table 6:** Comparison of average precision, recall, and F1-score under different methods.

Methods	ELM			RF			SVM		
	Average Precision	Average Recall	Average F1-score	Average Precision	Average Recall	Average F1-score	Average Precision	Average Recall	Average F1-score
MSC	85.86%	85.00%	85.43%	87.53%	87.50%	87.39%	85.51%	85.50%	85.50%
SG	87.88%	87.00%	87.44%	83.88%	84.05%	83.93%	85.01%	85.00%	84.96%
SNV	89.90%	89.00%	89.45%	87.04%	87.05%	87.03%	85.59%	85.50%	85.49%
MSC-IAO	85.58%	89.00%	87.25%	90.47%	90.45%	90.46%	73.53%	73.50%	73.50%
SG-IAO	86.54%	90.00%	88.24%	82.32%	82.35%	82.33%	84.06%	84.00%	84.00%
SNV-IAO	87.13%	88.00%	87.56%	86.95%	87.00%	86.90%	88.06%	88.00%	88.00%
MSC-EIAO	89.00%	89.00%	89.00%	91.46%	91.45%	91.45%	73.01%	73.00%	72.99%
SG-EIAO	90.82%	89.00%	89.00%	87.45%	87.45%	87.45%	80.53%	80.50%	80.50%
SNV- EIAO	90.00%	90.00%	90.00%	89.42%	89.45%	89.43%	90.54%	90.50%	90.49%

### 3.3 EIAO performs well on the UCI dataset

**Figure 6:**
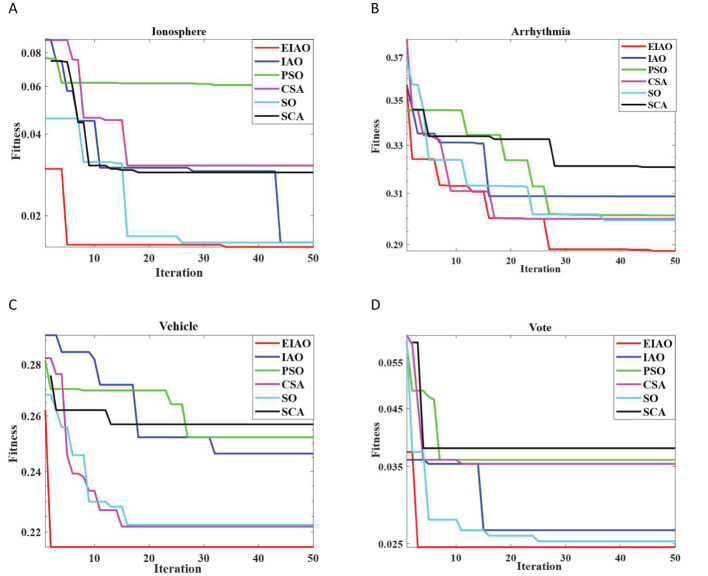
Stability analysis of EIAO and 5 baseline methods on 4 UCI datasets. **(A)** Ionosphere. **(B)** Arrhythmia. **(C)** Vehicle. **(D)** Vote.

To illustrate the generality of the algorithm, we tried to test EIAO and other five optimization algorithms on four UCI datasets. **Table 7** shows the average performance of EIAO and other five optimization algorithms on four UCI datasets. Due to the low feature dimension of the Vote dataset, the classification accuracy and the number of selected relevant features of the six algorithms are very close. On the Arrhythmia and Vehicle datasets, the classification performance of the algorithm is slightly inferior to that of the other two datasets. The reason may be that there are too many classification levels. For the Ionosphere dataset, the lowest optimal fitness value can be found, that is, the classification accuracy in Eq (5) is the highest and the least features are selected. At the same time, in order to analyze the robustness of EIAO, it is checked whether the convergence curve of the EIAO algorithm is stable in multiple repetitions and whether it converges quickly to the optimal solution in iterations. In **Figure 6**, it is observed that EIAO achieves good performance compared with the other five swarm intelligence algorithms.

**Table 7:** Performance comparisons between EIAO and 5 baseline algorithms on UCI datasets.

Metric	Algorithm	Ionosphere	Arrhythmia	Vehicle	Vote
Accuracy (%)	IAO	95.71	70.00	75.74	97.70
	EIAO	97.14	72.22	78.70	97.70
	CSA	94.29	67.78	78.11	96.55
	SCA	97.14	70.00	74.56	96.55
	PSO	95.71	65.56	75.15	96.55
	SO	95.71	70.00	78.11	97.70
Selected features	IAO	6	32	11	6
	EIAO	4	30	8	2
	CSA	13	122	9	3
	SCA	5	36	9	6
	PSO	10	108	11	3
	SO	3	74	10	4
Best fitness function	IAO	0.0159	0.3088	0.2463	0.0265
	EIAO	0.0153	0.2876	0.2153	0.0241
	CSA	0.0306	0.2998	0.2217	0.0354
	SCA	0.0289	0.3208	0.2569	0.0379
	PSO	0.0607	0.3012	0.2521	0.036
	SO	0.0159	0.2993	0.2223	0.0253

## 4 Discussion

Seed vigor is a crucial factor in crop cultivation and growth. Traditional methods for measuring vigor are often highly destructive and inefficient. Numerous studies have demonstrated that spectroscopy can effectively detect seed vigor parameters, while machine learning can be employed for modeling these parameters (Yang et al., 2017). For example, in chilling injury detection of eggplant, researchers have collected hyperspectral data that reflect internal physiological indicators and integrated them with machine learning to achieve consistent detection of eggplants stored under harmful cold conditions (Tsouvaltzis et al., 2020). This demonstrates that hyperspectral data can be used not only for quality evaluation but also hold great potential for monitoring changes in seed physiological status, thereby playing a significant role in improving the accuracy of seed vigor identification. While several studies have explored the integration of hyperspectral data with machine learning algorithms for the non-destructive analysis of seed quality parameters, research focused on vigor detection in this context remains limited. For instance, some studies have utilized hyperspectral data in conjunction with machine learning to assess the vigor of naturally aged rice seeds (Jin et al., 2022) (Xu et al., 2022). Another study combined visible and near-infrared hyperspectral data with machine learning techniques to non-destructively detect the vigor of watermelon seeds (Sun et al., 2024). These studies have provided preliminary evidence supporting the feasibility of using the full wavelength range for classifying seed vigor.

A series of feature selection algorithms were subsequently applied to extract characteristic wavelengths from the full spectral range, aiming to reduce data redundancy while preserving classification accuracy. In seed vitality studies using spectral detection, traditional wavelength screening methods such as SPA and CARS are commonly employed. However, these methods are prone to high sensitivity to noise and unstable selected bands. To address these issues, this study proposes a novel wavelength selection algorithm, EIAO, and applies it to the band selection problem. Compared to other wavelength screening methods, the EIAO algorithm not only reduced the number of selected features but also demonstrated superior performance in terms of classification accuracy. Specifically, EIAO achieved an accuracy of 92.0%, outperforming benchmark algorithms such as IAO, PSO, and SCA.

Furthermore, this study utilized three classifiers, ELM, RF, and SVM, combined with various wavelength selection methods for performance evaluation. As shown in the results presented in **Table 5**, wavelength screening and feature subset selection significantly enhanced classifier performance, particularly in the RF and SVM models. After wavelength screening optimized by EIAO, the accuracy of the RF model on the test set reached 91.45%, and the accuracy of the SVM model was 90.50%, both of which were substantially higher than those of the unscreened original data model (83.50% and 87.50%, respectively). These findings indicate that wavelength screening not only improves classification accuracy but also reduces unnecessary features, thereby enhancing model performance. This series of results underscore the importance of wavelength screening in hyperspectral data analysis, particularly in seed vigor classification. The improved accuracy following wavelength screening aligns with the findings of Bi (Bi et al., 2024).

The generalization capability of the EIAO algorithm is further evaluated in the final part of this study. Compared to other optimization algorithms, EIAO demonstrates strong classification performance across multiple datasets. Notably, on the Ionosphere dataset, the classification accuracy achieved by EIAO is 97.14%, which is significantly higher than that of IAO (95.71%) and other optimization algorithms. This result aligns with previous research (Desuky et al., 2021), where the performance of the newly proposed EAOA algorithm was validated on a dataset with 16 feature selections. In that study, the new algorithm achieved the highest classification accuracy in selecting the optimal feature subset from the training data.

## 5 Conclusions

This study investigates the potential of hyperspectral technology for the non-destructive detection of eggplant seed vitality. Hyperspectral data from the region of interest of eggplant seeds were extracted, and preprocessing algorithms, including MSC, SNV, and SG, were applied to reduce noise. The EIAO algorithm proposed in this study was then employed to select feature wavelengths, and classification models using ELM, RF, and SVM were constructed. The results demonstrated that data preprocessing and feature selection substantially enhanced classification performance compared to the original data, with the EIAO-based feature selection method yielding particularly favorable outcomes. The RF and SVM models exhibited strong generalization ability under the optimized features, especially on the test set. While the model based on the selected wavelengths resulted in the loss of some spectral information, it effectively reduced data redundancy and significantly improved classification accuracy. This study highlights the importance of selecting characteristic wavelengths for accurate modeling. Future research could further explore the integration of additional spectral information, such as texture features, to further enhance model performance.

## Data Availability

The raw data supporting the conclusions of this article will be made available by the authors, without undue reservation.
